# Assessing the genetic diversity in *Argopecten nucleus* (Bivalvia: Pectinidae), a functional hermaphrodite species with extremely low population density and self‐fertilization: Effect of null alleles

**DOI:** 10.1002/ece3.6080

**Published:** 2020-04-02

**Authors:** Judith Barros, Federico M. Winkler, Luz Adriana Velasco

**Affiliations:** ^1^ Laboratorio de Moluscos y Microalgas Universidad del Magdalena Santa Marta Colombia; ^2^ Departamento de Biología Marina Facultad de Ciencias del Mar Universidad Católica del Norte Coquimbo Chile; ^3^ Centro de Innovación Acuícola AquaPacífico Coquimbo Chile; ^4^ Centro de Estudios Avanzados en Zona Áridas (CEAZA) Coquimbo Chile

**Keywords:** Caribbean Sea, inbreeding, microsatellite, scallop, segregation analysis

## Abstract

*Argopecten nucleus* is a functional hermaphroditic pectinid species that exhibits self‐fertilization, whose natural populations have usually very low densities. In the present study, the genetic diversity of a wild population from Neguanje Bay, Santa Marta (Colombia), was estimated using microsatellite markers, and the effect of the presence of null alleles on this estimation was assessed. A total of 8 microsatellite markers were developed, the first described for this species, and their amplification conditions were standardized. They were used to determine the genotype of 48 wild individuals from Naguanje Bay, and 1,010 individuals derived from the offspring of 38 directed crosses. For each locus, the frequencies of the identified alleles, including null alleles, were estimated using the statistical package *Micro‐Checker*, and the parental genotypes were confirmed using segregation analysis. Three to 8 alleles per locus with frequencies from 0.001 to 0.632 were detected. The frequencies of null alleles ranged from 0.10 to 0.45, with *Ho* from 0.0 to 0.79, and *He* from 0.53 to 0.80. All loci were in H‐W disequilibrium. The null allele frequencies values were high, with lower estimations using segregation analysis than estimated using *Micro‐Checker*. The present results show high levels of population genetic diversity and indicate that null alleles were not the only cause of deviation from H‐W equilibrium in all loci, suggesting that the wild population under study presents signs of inbreeding and Wahlund effect.

## Graphical abstract

The microsatellite markers exhibited high levels of genetic diversity in the *Argopecten nucleus* population of Neguanje Bay (Santa Marta, Colombia), but also indicated a high homozygosity, suggesting the occurrence of the self‐fertilization associated with the low‐population densities reported for the species.

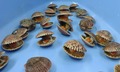

## 1 INTRODUCTION

*Argopecten nucleus* is a pectinid species endemic of the Caribbean Sea that forms very sparse populations in nature, to the extent that no natural beds have been described in literature (Díaz & Puyana, 1994; Lodeiros, Freites, Núñez, & Himmelman, 1993). Therefore, the only source of wild live specimens to date has been the obtention of seeds using artificial collectors suspended in the sea (Castellanos & Campos, 2007; Lodeiros et al., 1993). This species inhabits seagrass beds and sand bottoms, particularly those located in coral reefs zones, from 5 to 50 m of depth (Díaz & Puyana, 1994; Lodeiros et al., 1993). *Argopecten nucleus* is a species with a short life span (1–2 years), characterized by early sexual maturity (at 3 months) (Lodeiros & Freites, 2008; Velasco & Barros, 2008) and spawning activity occurring throughout the year (Lodeiros et al., 1993; Velasco, Barros, & Acosta, 2007). This species is also a simultaneous hermaphrodite with external fertilization that is able to release gametes of one sex first, usually male, before releasing other sex gametes, with a time lapse of approximately 15 min between them (Velasco et al., 2007).

Studies on population genetic structure of marine species attract considerable interest, since they allow us to assess the state of conservation of natural populations (Piñero, Caballero‐Mellado, Cabrera‐Toledo, & Zúñiga, 2008; Smith, 1996), to adopt proper management practices in their fisheries, and to develop aquaculture strategies to reduce the risk of inbreeding depression and fitness loss (Gjedrem & Baranski, 2009), thus contributing to sustainable production over time (Petersen, Baerwald, Ibarra, & May, 2012; Wang, Fu, & Xia, 2013). To date, there is no information about the genetic structure of wild populations of *A. nucleus*, but the low population densities usually found for this species suggest the existence of high levels of genetic drift and inbreeding, as previously reported for other mollusks such as *Haliotis iris* (Smith & Conroy, 1992). This situation could be further exacerbated due to the effects of spontaneous self‐fertilization, which has been estimated at 12% ± 1% on average for reproduction in hatchery (Barros, Velasco, & Winkler, 2018; Barros, Winkler, & Velasco, 2018). In *Argopecten irradians irradians*, it has been estimated that self‐fertilization might be able to reduce the genetic diversity of the population in 10%–40% in just one generation (Zheng, Zhang, Guo, & Liu, 2008). However, although similar or even higher self‐fertilization rates have been observed in *A. purpuratus* (Winkler & Estévez, 2003), no significant deviations from the Hardy–Weinberg (H‐W) equilibrium for allozyme loci have been observed in wild populations, and when present, deviations only affect some loci, but not all of them (von Brand & Kijima, 1990; Galleguillos & Troncoso, 1991; Moraga et al., 2001).

The use of selectively neutral genetic markers allows to estimate genetic diversity and obtain information about the genetic structure of populations, which can be useful to infer the occurrence of phenomena such as genetic drift and inbreeding (Blouin, 2003; Qin, Liu, Zhang, Zhang, & Guo, 2007; Wu, Feng, Ma, Pan, & Liu, 2015). Furthermore, they are valuable to understand the factors regulating population dynamics (Bert et al., 2014). Among these markers, microsatellites (SSR, Simple Sequence Repeats) are particularly convenient because they usually display Mendelian inheritance and codominance. SSRs are short DNA sequences of 2–6 base pairs that can repeat in tandem a variable number of times in the genome and are frequently flanked by sequences with unique copies in the genome, which make possible to design specific primers and to amplify them using polymerase chain reaction (PCR; Liu & Cordes, 2004; Tagu & Moussard, 2003). They exhibit high polymorphism and allele number per locus, and since they are noncoding sequences, their variations usually are not affected by natural selection (Liu & Cordes, 2004). These markers have been very useful for paternity analysis in bivalves (Taris, Baron, Sharbel, Sauvage, & Boudry, 2005), for the assessment the diversity and population genetic structure (Wang et al., 2013), and for studies of linkage and genetic maps building (Petersen et al., 2012; Qin et al., 2007).

Although most microsatellite loci show random recombination, deviations from Mendelian proportions have been reported in experimental crosses for different species associated with the presence of null alleles (Baranski, Rourke, Loughnan, Austin, & Robinson, 2006; De Meeûs, 2018; Hedgecock, Li, Hubert, Bucklin, & Ribes, 2004; Li, Park, Kobayashi, & Kiima, 2003; Zhan et al., 2007). Null alleles appear when a mutation affects one of the union sites of the primers with the flanking zone of the microsatellite and impedes that it amplifies, and heterozygotes for them are then genotyped erroneously as homozygotes (Lewin, 2004). The existence of null alleles can lead to errors in the estimation of allele frequencies, thus overestimating homozygosis (Becquet, Lanneluc, Bouhet, & García, 2009; Pereira, Arias, Méndez, Insua, & Freire, 2009; Weetman, Hauser, Shaw, & Bayes, 2005) and the inbreeding levels of a population (Jones, Stockwell, Walker, & Avise, 1998; Xu et al., 2001). It also affects the reliability of the use of microsatellite markers for other applications, such as paternity testing, estimation of the degree of relatedness between individuals, and decisions about species management, among others (Ardren, Borer, Thrower, Joyce, & Kapuscinski, 1999).

In order to correct the bias that the presence of null alleles can introduce in population genetic analysis, their frequency can be inferred using different theoretical models (Jones et al., 1998; van Oosterhout, Weetman, & Hutchinson, 2006; Xu et al., 2001). For such purposes, statistical packages based on maximum likelihood algorithms use the differences between the observed and the expected heterozygosity in a sample to estimate their frequencies (Brookfield, 1996; Chakraborty, de Andrade, Daiger, & Budowle, 1992; van Oosterhout, Hutchison, Wills, & Shipley, 2004). Software packages such as *Micro‐checker* indicate the presence of null alleles if the combined probability of Fisher test shows a significant, general excess of homozygotes that are evenly distributed between the homozygotes classes (van Oosterhout et al., 2004). The presence of null alleles has been observed in different aquatic species, such as the bivalves *Donax trunculus* (Rico et al., 2017), *Pinna nobilis* (González‐Wangüemert et al., 2015), *Pinctada margaritifera* (Lemer, Rochel, & Planes, 2011) and *C rassostrea gigas* (Hedgecock et al., 2004), and the flatfish *Scophthalmus maximus* (Borrell et al., 2004), among others. These estimations, however, do not allow to correct the index of genetic diversity, and the precision and confidence interval of the estimations can be very variable depending on the real frequency of the null alleles and other factors such as the sample size, the number of sampled generations, the level and duration of the genetic bottlenecks of the population, nonrandom mating, changes of density or migration rates (Dabrowski et al., 2014). In addition, the frequencies of null alleles could be overestimated due to the occurrence of inbreeding (Chybicki & Burczyk, 2009).

An alternative method to estimate the actual frequency of null alleles is the use of segregation analysis, a method that allows to verify the presence of recessive alleles in the genotype of individuals with dominant phenotype, based on data from families (Elston, 1981). Under an appropriated crossing design, it can be used to verify the genotype of a putatively homozygous individual for a dominant gene, based on the genotypes of their progenies, and the expected frequencies with respect to the corresponding Mendelian proportions (Zhan et al., 2007). This kind of analysis has been used to test the presence of null alleles in the Pacific oyster (*Crassostrea virginica*; Reece, Ribeiro, Gaffney, Carnegie, & Allen, 2004), rainbow trout (*Oncorhynchus mykiss*; Holm, Loeschcke, & Bendixen, 2001), the Coleoptera *Pissodes strobi* (Liewlaksaneeyanawin, Ritland, & El‐Kassaby, 2002), hop (*Humulus lupulus*; Vašek et al., 2010) and coffee (*Coffea liberica*; López, Rutherford, & Moncada, 2014), among other species. Therefore, the segregation analysis allows to correct the biases generated by the presence of null alleles on microsatellite markers, thus making the estimations of genetic diversity more reliable when such alleles are present in high frequencies in a population.

At present, there are no microsatellite markers described for *A. nucleus*, and hence, this tool is not currently available to study relevant genetic aspects of its populations. In addition, there is no information on the genetic diversity of this scallop species in natural populations. Therefore, the purpose of the present study is to identify and characterize the first set of primers for microsatellite loci in *A. nucleus* and use them to estimate the genetic diversity in a wild population from Bahía Neguanje, Santa Marta (Colombia), as well as to assess the effect of the presence of null alleles on the estimations of genetic diversity in a species with self‐fertilization and very low population densities in the wild.

## 2 MATERIALS AND METHODS

### 2.1 Experimental design

To evaluate the levels of genetic diversity in *A. nucleus*, primers for 8 SSR loci were developed. At the same time, scallops from a wild population were collected and conditioned in captivity. They were later induced to spawn under controlled conditions, performing cross‐fertilization following a nested design. Observed self‐fertilization rates were registered during spawning process for each individual used as dam. The progeny of each full‐sib family was cultured separately until they reach adulthood. Tissue samples were extracted from each parent and their progenies for the microsatellite loci genotyping. The genotype of each parent was confirmed after the segregation analysis of the SSR alleles in their progenies.

### 2.2 Microsatellites obtaining

#### 2.2.1 Obtention of biological material and DNA

In order to develop and standardize the microsatellites, samples of approximately 0.1 cm^3^ were obtained from the abductor muscle of 10 adults of *A. nucleus* (43.6 ± 4.3 mm shell length), which were produced by cross‐fertilization in the Laboratorio de Moluscos y Microalgas of Universidad del Magdalena, and cultured in suspended systems in Taganga Bay (Santa Marta) (11°16′03″N, 74°11′24″W). Tissue samples were preserved in ethanol 99% at room temperature until their analysis. DNA was extracted using the phenol–chloroform technique (Gardes & Bruns, 1993), until obtaining 20 µl with a concentration of 50 ng/µl of DNA. DNA integrity was confirmed using electrophoresis in agarose gel 1.2% and stained with SYBR Green. The amount of DNA was quantified in an Epoch equipment, and its purity was verified by the absorbance ratio 260/280 nm, using DNA samples with 1.8–2.0. The DNA was sent to the OMICS‐Solutions company, which provided a genomic library enriched with 300 microsatellites (SSRs) and their respective flanking regions. The microsatellite reads were characterized based on the number of nucleotides of their motif, structure, and the number of repeats.

#### 2.2.2 Microsatellite standardization

From the library, 10 microsatellite loci were selected based on (a) the repetition motif composed of 3 no degenerate bases; (b) the expected products of the PCR amplification in a range between 150 and 350 base pairs (bp); and (c) the expected annealing temperature in a range between 55 and 60°C. Pairs of primers flanking microsatellite sequences (F and R) were designed using the Primer3web v4.1.0 software (Kõressaar et al., 2018) and tested. Microsatellite loci with clearly distinct amplification products and consistent amplification were selected for the study. The conditions of PCR amplification were standardized for the 10 loci, testing different annealing temperatures. An initial denaturation temperature of 95°C for 5 min was used, followed by 30 denaturation cycles at 94°C for 45 s, an annealing gradient (Ta) of 46 to 56°C for 30 s (Table 1), extension at 72°C for 45 s, and finally 5 min at 72°C (Orozco & Narváez, 2014). These reactions were performed in a gradient thermocycler ESCO‐SWIF MaxPro, with a final volume of 10 μl of reaction mixture; 1.5 μl DNA with a concentration of 5 ng/µl, 2 μl reaction buffer (20 mM Tris‐HCl; pH 8; 50 mM KCl, final), 0.3 μl MgCl_2_ (1.5 mM), 0.2 μl dNTPs (0.2 mM), 0.2 μl of each primer (0.2 μM), and 1 μl Taq polymerase (0.5 U, Biolase©). PCR products were separated by capillary electrophoresis in a QIAxcel Advanced equipment, QIAGEN, using the High Resolution Kit QIAGEN and a ladder of 50–800 bp (v2.0 QIAGEN). These general conditions were used for PCR in the following amplifications, with adjustments of annealing temperature for each locus, as shown in Table 1.

**Table 1:** Characteristics of the 8 microsatellite loci isolated in *Argopecten nucleus* from Neguanje Bay, Colombia

Locus	Repeated motif	Primers sequences Forward (F) and Reverse (R)	Ta (°C)	Size range (bp)	PIC
*An001*	(ATA)_n_	F: ATTCCTATGTACGTCATGTC R: CATGAAGATACCTCTTACAG	50.3	119–134	0.68
*An002*	(AAC)_n_	F: CTGTATGTAAAGCACAGATG R: ACCGACTTAAGATGTATGTC	50.3	173–188	0.63
*An003*	(ATA)_n_	F: ATTCCTATGTACGTCATGTC R: CATGAAGATACCTCTAACAG	50.3	119–131	0.69
*An005*	(CAT)_n_	F: GAATATTAACCAGACCAGAG R: CAACAGTACTTACATGTTCG	50.3	162–174	0.50
*An006*	(TAA)_n_	F: ATTCCTATGTACGTCATGTC R: ACACTCTTCAACGTTTACAC	50.3	172–190	0.70
*An007*	(ATA)_n_	F: CCAGTAGTTTCTGAGAAAAG R: CATGAAGATACCTCTAACAG	52.3	128–149	0.75
*An009*	(ATG)_n_	F: ACATCTCCTTGTACCCTATAC; R: AGACTTTCAAGAGACACTCTC	50.3	125–134	0.64
*An010*	(TTG)_n_	F: TCTAACCTGTCCATCATATC; R: GACAATGAAACAGGTATCAC	51.0	153–159	0.44

Abbreviations: PIC, polymorphic information content; Ta, annealing temperature.

### 2.3 Segregation analysis

From crosses of 48 wild scallops (41.2 ± 1.8 mm shell length) collected from Neguanje Bay, Santa Marta (Lat. 11°20′03′′N, Long. 74°09′24′W), a total of 1,010 individuals were obtained from 38 full‐sib and 10 half‐sib families. Brooders were obtained as seed from artificial collectors suspended in the bay area, which were provided by the Instituto de Investigaciones Marinas y Costeras (INVEMAR, Santa Marta). Families were built as described by Barros, Velasco, et al. (2018), Barros, Winkler, et al. (2018), following the protocols for spawning and culture described by Velasco and Barros (2007, 2009) and Velasco et al. (2007). Crosses were performed using a nested design, according to which the sperm of one scallop used as male (sire) was used to separately fertilize the oocytes of 2–4 individuals (dams). Thus, each dam was crossed with only one male and no scallop was simultaneously used as mother and father. The self‐fertilization rate was estimated as the frequency of nonfertilized oocytes that spontaneously initiate embryonic development within 4 hr after spawning in a sample (10 ml) from each individual used as female (Barros, Velasco, et al., 2018; Barros, Winkler, et al., 2018; Winkler & Estévez, 2003).

When the progenies were 9 months old, the scallops were removed from the culture system and samples of the mantle tissue (aprox. 0.05 cm^3^) were collected (60 scallops per FS‐family). Similar mantle tissue samples were obtained by biopsy from the 48 available brooders. Samples were individually fixed and preserved in ethanol 99% at room temperature until DNA extraction. DNA was extracted following the methods of Lopera‐Barrero et al. (2008), replacing sodium chloride for ammonium acetate*.* DNA integrity was confirmed using agarose gel (0.8%) electrophoresis (80 V, 40 min) stained with GelRed, and visualized in a Biodoc UVP LLC transilluminator.

To estimate the genetic diversity of the population of Neguanje Bay, the genotype from each individual was assessed for the microsatellite loci previously standardized. The putative genotype of each parent was confirmed based on the genotypes observed in the offspring of each mating. The existence of full‐sib and half‐sib families allowed to confirm the genotype of each father in two or more crosses with different mothers, thus reducing the uncertainty in determining genotypes that can be introduced by the potential occurrence of self‐fertilization. An individual was considered to be the result of self‐fertilization when its genotype for 2 or more loci analyzed was only possible to arise if both alleles came from the individual used as mother, and the genotype for the remaining loci did not contradict this hypothesis. For this analysis, absence of self‐fertilization in the fathers was assumed. Therefore, in crosses between two putative homozygotes for different alleles that progenies presented three genotypes, both parents were assumed to be heterozygous for a null allele, regardless of the genotypic proportions in the progeny of that cross.

### 2.4 Data analysis

The size of each amplification product from individuals used as broodstock, and their progeny were determined with the software QIAGEN ScreenGel QIAxcel v1.0. The polymorphic information content (PIC) was estimated with the software CERVUS 3.0.7. For the wild brooders, the null allele frequencies were estimated using the models of van Oosterhout (Girard & Angers, 2008), Brookfield 1 (Brookfield, 1996), and Brookfield 2 (Girard & Angers, 2008) methods using the *Micro‐Checker* 2.2.3 package. The allelic frequencies, as well as the number of alleles (*Na*), observed (*Ho*) and expected (*He*) according to the H‐W equilibrium heterozygosities, *F_IS_* statistic, and deviations of the H‐W equilibrium were estimated with the GenAlEx 6.4 software (Peakall & Smouse, 2009). The allelic frequencies were corrected according to the frequency of null alleles estimated by the Brookfield 1 model, and the standard deviation of allelic frequencies per locus was calculated as $SD=\sqrt{\left(1-\Sigma p_{i}^{2}\right)/\left(2N\right)}$, where $p_{i}$ is the frequency of the *i*th allele in the population and *N* is the sample size (Cockerham, 1969). For data from the segregation analysis, observed (*Ho*) and expected (*He*) heterozygosity, the frequency of null alleles, and *F*_IS_ were estimated according to Nei (1975).

The estimations of the different genetic parameters obtained for wild (= parental) animals with the two methods for detection of null alleles were compared using a one‐way ANOVA, followed by Tukey's post hoc test for multiple comparisons. The normality and homoscedasticity of all the mentioned response variables were previously examined by the Kolmogorov–Smirnov's and the C of Cochran tests, respectively. In the case of *He,* this parameter was analyzed using the nonparametric test of Kruskal–Wallis, since data did not comply with the parametric requirements of homoscedasticity and normality (Zar, 1999). The consistency of the frequencies of null alleles estimated with both methods was verified using the Spearman correlation analysis. All the statistical analyses were performed using the software Statgraphics Centurion XVI.I.

## 3 RESULTS

From the 10 primer pairs selected, two pairs were not included in the analysis since they could not be consistently amplified by PCR, even when using different annealing temperatures and reactants concentrations. The remaining 8 primer pairs exhibited reproducibility and consistent amplification over time. The designation of each locus, the motif of each microsatellite, and the sequences of the primer pairs (forward and reverse) are detailed in Table 1, in addition to the conditions for optimal PCR amplification, the size range of the alleles in each locus, and the polymorphic information content (PIC).

Between 0% and 25% of the zygotes derived from each family exhibited embryo development in unfertilized oocytes samples, with an average of 12% ± 7%. From 304 potential genotype combinations per locus, only 255 were observed and 214 of them were useful to infer the probable genotype of the parents. The remaining 41 genotype combinations were not informative, since both putative parents and their progenies were phenotypically homozygotes, or the number of individuals recovered per family was too low to verify the Mendelian proportions (see Appendix S1). In 76% of the families, allele segregation was observed for all markers. In 8 of the 38 families analyzed (21%), 1 to 5 individuals (4%–20%) exhibited a genotype that indicated the occurrence of self‐fertilization, that is, the genotype for more than two loci could only arise from the fusion of gametes produced by the individual used as mother.

**Figure 1:**
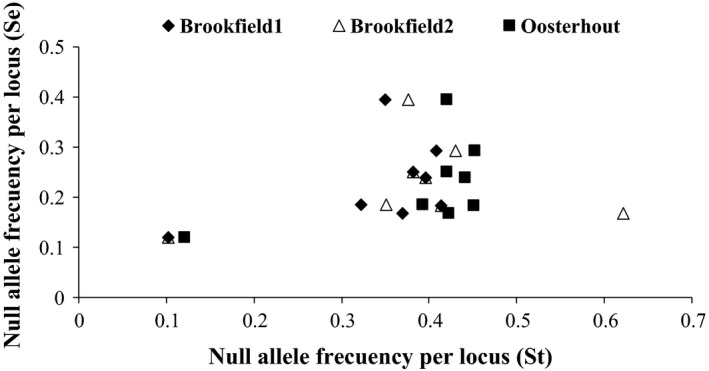
Association between the null allele frequency estimations using segregation analysis (Se) and Micro‐Checker 2.2.3 software (St) applying three different models

All loci were polymorphic in the wild population (= parental), with 4 (*An010*) to 9 (*An007*) alleles per locus (Appendix S1). The allelic frequencies ranged from 0.005 (*An001*) to 0.313 (*An007*) and were similar to the range of frequencies obtained using the software *Micro‐Checker* 2.2.3 and from the segregation analysis with similar standard deviations of allele frequencies using both methods (Table 2). The frequencies of null alleles obtained from the segregation analysis ranged from 0.10 to 0.41 (Table 2), and the estimations inferred using *Micro‐Checker* 2.2.3 were, on average, 11.5% ± 9.8% higher than those estimated based on the segregation analysis (*p* < .05, Table 2). No significant correlation across loci was observed between both methods for the estimation of null allele frequencies (Figure 1) (*r* < .3114; *p* > .05). There were no statistical differences between null allele frequencies estimated using different models (van Oosterhout, Brookfield 1 and 2) (*p* > .05). The observed heterozygosity (*Ho*) ranged between 0 and 0.79, depending on the locus and estimation method for allele frequencies, while the expected heterozygosity (*He*) ranged from 0.53 and 0.80, with higher values obtained based on the segregation analysis (*p* < .05, Table 3). The values of *F*_IS_ ranged between −0.01 and 1.00, with lower values estimated based on the segregation analysis (*p* < .01, Table 3).

**Table 2:** Comparison of allelic frequencies in 8 microsatellite loci in wild (= parental) individuals of *Argopecten nucleus* from Neguanje Bay, Colombia*,* estimated using Micro‐Checker 2.2.3 software (St) and segregation analysis (Se). 0: null allele

Loci	*N*	Allelic frequencies											*SD*
										Brookfield1 (Used in this study)	Oosterhout	Brookfield2	
*An001*		*119*	*122*	*125*	*128*	*131*	*134*			*0*	*0*	*0*	
St	40	0.072	0.242	0.108	0.176	0.015	0.005			0.382	0.421	0.382	0.097
Se	40	0.125	0.188	0.138	0.263	0.025	0.013			0.250			0.100
*An002*		*173*	*176*	*179*	*182*	*185*	*188*			*0*			
St	40	0.010	0.020	0.169	0.266	0.118	0.020			0.397	0.441	0.396	0.095
Se	40	0.013	0.038	0.250	0.288	0.150	0.025			0.238			0.098
*An003*		*119*	*122*	*125*	*128*	*131*				*0*			
St	30	0.109	0.203	0.089	0.169	0.015				0.415	0.451	0.414	0.111
Se	30	0.250	0.250	0.100	0.167	0.050				0.180			0.116
*An005*		*162*	*165*	*168*	*171*	*174*				*0*			
St	38	0.034	0.169	0.405	0.039	0.017				0.336	0.393	0.351	0.095
Se	38	0.053	0.211	0.434	0.079	0.039				0.184			0.098
*An006*		*175*	*178*	*181*	*184*	*187*	*190*			*0*			
St	36	0.029	0.133	0.268	0.059	0.059	0.039			0.414	0.423	0.622	0.101
Se	36	0.056	0.208	0.333	0.083	0.069	0.042			0.167			0.106
*An007*		*128*	*131*	*134*	*137*	*140*	*143*	*146*	*149*	*0*			
St	42	0.008	0.288	0.242	0.030	0.061	0.121	0.106	0.038	0.106	0.121	0.102	0.099
Se	42	0.150	0.313	0.284	0.045	0.104	0.030	0.149	0.060	0.119			0.094
*An009*		*125*	*128*	*131*	*134*					*0*			
St	29	0.186	0.128	0.226	0.49					0.411	0.453	0.431	0.092
Se	29	0.259	0.190	0.121	0.138					0.290			0.116
*An010*		*153*	*156*	*159*						*0*			
St	18	0.271	0.346	0.033						0.350	0.421	0.377	0.138
Se	18	0.242	0.242	0.121						0.394			0.141

Abbreviations: N, number of individuals analyzed; *SD*, standard deviation of allelic frequencies per locus.

**Table 3:** Comparison of genetic diversity estimations in 8 microsatellite loci in wild (= parental) individuals of *Argopecten nucleus* from Neguanje Bay, Colombia, using the *Micro‐Checker* 2.2.3 software (St) and segregation analysis (Se) to estimate null allele frequencies

Locus	*Ho*		*He*		*F*_IS_	
	St	Se	St	Se	St	Se
*An001*	0.07	0.55	0.73	0.80	0.90*	0.31
*An002*	0.02	0.50	0.69	0.77	0.98*	0.35
*An003*	0.02	0.40	0.74	0.80	0.98*	0.50
*An005*	0.04	0.42	0.53	0.72	0.93*	0.42
*An006*	0.02	0.36	0.62	0.80	0.96*	0.55
*An007*	0.60	0.79	0.79	0.78	0.23*	−0.01
*An009*	0.00	0.59	0.70	0.78	1.00*	0.25
*An010*	0.00	0.72	0.54	0.71	1.00*	−0.01
*Media*	0.10^b^	0.54^a^	0.68^b^	0.77^a^	0.87^a^	0.29^b^

Note

Different super indexes indicate significant differences between both methods (*p* < .05).

Abbreviations: *Ho*, Observed heterozygosity; *He*, Expected heterozygosity; *F*_IS_, Homozygosity Index.

* H‐W *p* < .01.

## 4 DISCUSSION

The present study reports the first 8 microsatellite markers for *A. nucleus,* which were designed, standardized, and used to estimate the genetic diversity in a wild population of this species. This population exhibited relatively high values of genetic diversity, although a considerable excess of homozygotes with respect to Hardy–Weinberg equilibrium was observed. The segregation analysis shows that *Micro‐Checker* 2.2.3 tends to overestimate the null allele frequencies in comparison with the segregation analysis, thus generating a bias that was not consistent across loci.

The intrapopulation genetic diversity of wild organisms for neutral loci, such as microsatellites, is mainly driven by the counteracting effects of genetic drift and inbreeding, which tend to reduce it, and the effects of mutation and migration, which are able to increase it (Gjedrem & Baranski, 2009). The wild population of *A. nucleus* from Neguanje Bay exhibited high levels of genetic diversity, in terms of polymorphism and number of alleles per locus. These results are similar to those encountered in wild populations of other species of bivalve mollusks using this kind of markers, such as *M. chilensis* (Larraín et al., 2015), *M. galloprovincialis* (Li, Liang, Sui, Gao, & He, 2011), *Cerastoderma edule* (Martínez, Arias, Méndez, Insua, & Freire, 2009) and *Pinna nobilis* (González‐Wangüemert et al., 2015), and also similar to other pectinids such as *A. purpuratus* (Marín, Fujimoto, & Arai, 2013), *C. nobilis* (Hui et al., 2006), *Patinopecten yessoensis* (Li, Qi, Nie, Kong, & Yu, 2016), and *N. subnodosus* (Ibarra, Petersen, Famula, & May, 2006).

The deviations from Hardy–Weinberg equilibrium observed for all loci on the studied wild population shows a considerable excess of homozygotes, which can be attributed to several factors. One of those factors is the presence of null alleles, which are caused by a failure in the microsatellites amplification during PCR (Callen et al., 1993; Stadhouders et al., 2010; Wattier, Engel, Saumitou‐Laprade, & Valero, 1998). They are particularly frequent in marine invertebrates (Hare, Karl, & Avise, 1996; Hedgecock et al., 2004), and their occurrence can result in the incorrect classification of heterozygote individuals for these alleles as homozygotes for the dominant alleles (Dakin & Avise, 2004; Lemer et al., 2011; Pompanon, 2005). Other factors that can further reduce the observed frequency of heterozygotes in a population are high levels of inbreeding (Chapuis & Estoup, 2007) and the Wahlund effect (Wahlund, 1928).

The use of statistical tools and the segregation analysis evidenced the presence of null alleles in all the examined microsatellite loci in *A. nucleus*, with similarly high accuracy, which can significantly influence the estimated values of heterozygosity and inbreeding in this species. The frequency of null alleles per locus was high (39%–45%), with the exception of locus *An007* (12%). Their frequency estimated using statistical methods, however, was higher than those obtained from the segregation analysis, and no significant correlation between the results using both methods was observed. The segregation analysis of alleles allows to infer the parental genotype based on the parental phenotype and the genotypes observed in their offspring (Reece et al., 2004). Although this analysis is methodologically more laborious, it is a direct and reliable technique to estimate the frequency of null alleles in a population. However, if both parents are phenotypically homozygotes for the same dominant allele, it is not possible to infer with certainty if one or both parents are heterozygotes for a null allele with the same genotype. Thus, the frequency of the null alleles estimated using this method could be slightly underestimated. The mating of one male with several females reduces this risk by increasing the certainty about the genotypes of the males and improving the reliability of the inferences about the mother's genotype. However, it does not completely prevent the difficulties that arise from determining the genotype of the fathers, as it has been observed in individuals (20%) whose genotype could not be confirmed for some loci using this method in the present study. In addition, if the individuals of *A. nucleus* used as parents were able to contribute to the offspring by self‐fertilization, this technique could overestimate the frequencies of null alleles.

The estimation of the frequency of null alleles using statistical methods, such as those included in softwares like *Micro‐Checker* v.2.2.3 (van Oosterhout et al., 2004; Shipley, 2003), and GENEPOP v.3.4 (Raymond & Rousset, 1995), are based on the assumptions that the presence of null alleles produces an excess of homozygotes in the population dataset, in comparison with the expected Hardy–Weinberg equilibrium (van Oosterhout et al., 2004). Both inbreeding and the Wahlund effect can cause a consistent increase of homozygosity across the genome, unlike the effect of the null alleles, whose influence varies among loci depending on the frequencies of null alleles present in them (Girard & Angers, 2008; Waples, 2015). Nonetheless, the consequences of null alleles, inbreeding or the Wahlund effect are rather complex to distinguish solely based on the application of statistical analysis strategies. Recently, different tools for statistical inference have been proposed to distinguish between the presence of null alleles and the Wahlund effect in studies on population genetics (De Meeûs, 2018; Waples, 2015, 2018; Zhivotovsky, 2015), but their application requires the use of specific sampling designs (De Meeûs, 2018; Waples, 2018) and does not consider the simultaneous occurrence of inbreeding. The estimations of null allele frequencies in the wild population of *A. nucleus* using statistical methods were much higher than those obtained from the segregation analysis, and no significant correlation was found between them. These results are not in agreement with those reported in the study by Oddou‐Muratorio, Vendramin, Buiteveld, and Fady (2009), in which no significant differences were found between the use of segregation analysis and statistical methods for the tree species *Fagus sylvatica*, suggesting that frequency estimations for null alleles with both methods could strongly depend on the studied species, probably as a consequence of its reproductive strategy and population structure. Thus, the reproductive strategy and population structure must be considered when defining the experimental design for analyzing the genetic diversity in species similar to *A. nucleus*. The use of alternative methods to statistical analysis for estimating null alleles frequency in those populations is highly recommended, as well as increasing the number of studied loci and eliminating those that present significantly high frequencies of null alleles (Bürkli, Sieber, Seppälä, & Jokela, 2017; De Sousa, Finkeldey, & Gailing, 2005; Estoup, Jarne, & Cornuet, 2002; Oddou‐Muratorio et al., 2009; Stadhouders et al., 2010); redesigning primers to avoid the presence of mutations affecting the primer pairing with flanking zones (Holm et al., 2001; Reece et al., 2004); and, whenever possible, performing a segregation analysis to infer the parental genotypes. Another option is the use of SNPs markers, since they are easier to identify, show Mendelian segregation, and exhibit few null alleles at controlled crosses, compared to microsatellites (Harney et al., 2018).

*Argopecten nucleus* is a functional hermaphrodite that forms sparse, low‐density populations in nature. To date, the only source of live wild individuals has been seeds obtained from artificial collectors suspended in the sea (0–19 seed m^−2^ collector) (Díaz & Puyana, 1994; Lodeiros et al., 1993; Valero et al., 2000; Velasco & Barros, 2009). The data registered during the controlled spawning process indicate that 0%–25% of zygotes per family began spontaneous embryonic development after the gametes release, with an average of 12%, presumably due to self‐fertilization. Such levels of selfing are similar to those reported for other species of hermaphroditic mollusks, like *A. purpuratus* (Toro, Montoya, Martínez, Gutiérrez, & Vergara, 2010; Winkler & Estévez, 2003) and *Radix balthica* (7%–20%; Bürkli et al., 2017) under similar experimental conditions, although in *A. purpuratus* the selfing rates can be very variable among different females (Concha, Figueroa, & Winkler, 2011; Toro et al., 2010; Winkler & Estévez, 2003). In addition, the occurrence of self‐fertilization has been verified by molecular analysis of massive spawns in farmed *A. irradians* (Li & Li, 2011) and *N. subnodosus* (Petersen, Ibarra, Ramirez, & May, 2008). Winkler and Estévez (2003) have hypothesized that self‐fertilization in *A. purpuratus* takes place in the nephridia during the release of gametes, since all possible precautions to avoid self‐fertilization of the released oocytes were considered in hatchery operations. The same situation seems to occur in *A. nucleus.*

The adults of *A. nucleus* exhibit a limited capability of displacement, but its larvae spend 11–15 days in the plankton. In the particular geographic area of this study, planktonic larvae are continuously exposed to diverging marine currents, such as the Caribbean current that flows toward the west and the Darien countercurrent that flows in direction to north (CIOH, 2018). In addition, low salinity barriers (0–38.3 ppt) existing in the mouths of rivers Magdalena, Canal del Dique, Sinú and Atrato (Vivas‐Aguas, Espinoza, Sánchez, Cadavid, & Ibarra, 2012) can act as natural barriers to larval dispersion near to the coast, since low salinities are lethal for this pectinid. As a consequence, it is possible that self‐fertilization might be the rule rather than the exception for *A. nucleus*, due to the occurrence of self‐fertilization during the gametes release, and considering that low densities of natural populations could imply a low chance of encounter between gametes released by different individuals. It has been estimated that oocytes must be fertilized within 2 hr after release, and for sperm, the time lapse would be 4 hr as maximum (Velasco, 2008). Thus, functional hermaphroditism and self‐fertilization could have been evolved as an efficient reproductive strategy for a species with extremely low population densities. As a result, the offspring of different crossing events would exhibit high levels of individual inbreeding, but allelic frequencies would remain quite diverse between crosses. Oceanographic factors, population structure, and reproductive strategy could generate conditions favoring high levels of homozygosis, which is in agreement with what has been observed in the population of Neguanje Bay, as a result of the added influence of inbreeding and Wahlund effect.

A singular aspect of the present results is the high frequency of null alleles in most of the loci. Kimura and Ohta (1969) inferred that the number of generations required for the fixation by chance of a new selectively neutral mutation in a finite population depends on the effective population size. As a consequence, the combination of low densities populations and selfing in *A. nucleus* could favor the accumulation of selectively neutral mutations in a particular population, as it occurs when the population size decreases (Kimura, 1979). Therefore, it can be inferred that if a set of new independent microsatellite markers is developed for this species, the frequency of null alleles per locus will likely be similar to those observed in this study.

Assuming that *A. nucleus* have populations with very low density and high rates of self‐fertilization, as the results of this study suggest, a remarkable feature is their high levels of polymorphism and allele richness. Genetic evidences suggest that individuals of *A. purpuratus* exhibiting more inbreeding have higher mortality rates than their less inbred sibs (Toro et al., 2010; Winkler et al., 2009). The same phenomenon could be occurring in *A. nucleus*, thus contributing to preserve the genetic variability in wild populations. On the other hand, in a hermaphroditic species with high fecundity and low chance of cross reproduction, the effective population size in time will tend to be one. This implies that within‐population genetic variability can be low, but total genetic variability could remain unaffected (Falconer & Mackay, 2006), although out of the H‐W equilibrium due to the Wahlund effect and inbreeding. Aquaculture usually induces changes in the populations genetic structure and diversity due to founder effects, low effective number (Ne) of brooders, differences in genetic contribution of brooders in the reproductive process and domestic selection (Hedgecock & Sly, 1990; Li, Shu, Yu, & Tian, 2007; Liu, Zeng, Du, & Rao, 2011; Praipue, Klinbunga, & Jarayabhand, 2010; Rhode et al., 2012; Verspoor, 1988, among others). As a consequence, the genetic pool of wild populations might be negatively affected when populations generated through aquaculture are used for restocking wild populations, or gametes admixture occurs due to both wild and aquaculture populations share the same environment (Beaumont, 2006; Harada, Yokota, & Iizuka, 1998; Ryman, Jorde, & Laikre, 1995; Ryman & Laikre, 1991; Waples, Hindar, Karlsson, & Hard, 2016). However, the present results in *A. nucleus* seem to represent a paradox in this sense, because even when aquaculture populations and artificial reproduction can reduce the inbreeding and increase the Ne in comparison with wild populations, both factors can cause genetic loss in wild populations subject to supportive breeding or exposed to genetic introgression from cultured populations. To minimize the potential genetic impact of hatchery‐produced scallops on wild populations, there are different alternatives, including the systematic use of wild brooders (Yokota, Harada, & Iizuka, 2003), the use of completely genealogized brooders to ensure low inbreeding during a controlled reproduction process (Evans, Bartlett, Sweijd, Cook, & Elliott, 2004), the use of genetic markers to avoid inbred crosses (Liu et al., 2011), and the culture of triploids to prevent reproduction and genetic introgression in wild populations (Piferrer et al., 2009). However, this last method is not completely safe if the triploidization is not complete or if the triploids are not completely sterile (Winkler, Concha, & Concha, 2019).

In summary, the first 8 microsatellites designed and standardized for *A. nucleus* are reported, all of which were polymorphic with 4–9 alleles per locus. The segregation analysis evidenced an overestimation of the null alleles frequencies using regular statistical tools, and a lack of correlation between this data and the direct estimation of segregating null alleles, suggesting that the use of both methods can introduce an important bias in the estimations of null allele frequencies in populations that are highly polymorphic but have very low observed heterozygosity. The microsatellite markers exhibited high levels of genetic diversity in the *A. nucleus* population of Neguanje Bay (Santa Marta, Colombia), but also indicated a high homozygosity, suggesting the occurrence of the self‐fertilization associated with the low population densities reported for the species.

## CONFLICT OF INTEREST

Authors declare that they have no conflict of interest.

## AUTHOR CONTRIBUTIONS

The author's contributions to this study were as follows: J. Barros performed the experiments, analyzed the data, and wrote this manuscript. L.A. Velasco provided the original idea, advised the experiments, and wrote this manuscript. F.M. Winkler advised the experimental design and analyses as well as wrote this manuscript.

## ETHICAL APPROVAL

All applicable international, national, and/or institutional guidelines for the care and use of animals were followed.

## Data Availability

Supporting information of microsatellite genotypes in Appendix S1: Dryad: https://doi.org/10.5061/dryad.h9w0vt4f0.
