# Supplementary material for: Assessing the genetic diversity in Argopecten nucleus (Bivalvia: Pectinidae), a functional hermaphrodite species with extremely low population density and self‐fertilization: Effect of null alleles
Source: Ecol Evol. 2020 Apr 2;10(9):3919–31. doi: 10.1002/ece3.6080 (PMC7244797; doi:10.1002/ece3.6080)
Supplement: Supplementary file 1 [file ECE3-10-3919-s001.docx]

Appendix

| Family | Locus | Fenotype | | Frequency (F) in each genotypic class | | | | | | | | | | Sub Total | NA | Total | Potential parents (= wild) genotypes | | |  |
| --- | --- | --- | --- | --- | --- | --- | --- | --- | --- | --- | --- | --- | --- | --- | --- | --- | --- | --- | --- | --- |
|  |  | Sire | Dam | Class 1 | F 1 | | Clase 2 | F 2 | | Clase 3 | | F 3 | |  |  |  | Sire | Dam | |  |
| 2 | 1 | 122/134 | 122/122 | 122/122 | 2 | | 122/134 | 4 | | 134/134 | | 2 | | 8 |  | 8 | 122/134 | 122/0 | |  |
| 2 | 2 | 185/185 | 179/185 | 185/185 | 4 | |  |  | | 179/179 | | 4 | | 8 |  | 8 | 185/0 | 179/185 | |  |
| 3 | 2 | 179/179 | 182/182 | 179/179 | 10 | | 179/182 | 1 | | 182/182 | | 36 | | 47 |  | 47 | 179/0 | 182/0 | |  |
| 3 | 3 | 119/119 | 122/122 | 119/119 | 5 | | 119/122 | 15 | | 122/122 | | 23 | | 43 |  | 43 | 119/0 | 122/0 | |  |
| 3 | 5 | 165/165 | 171/171 | 165/165 | 9 | | 165/171 | 23 | | 171/171 | | 16 | | 48 |  | 48 | 165/0 | 171/0 | |  |
| 3 | 9 | 128/128 | 125/125 | 128/128 | 20 | | 128/125 | 24 | | 125/125 | | 3 | | 47 |  | 47 | 128/0 | 125/0 | |  |
| 3 | 10 | 156/156 | 153/153 | 156/156 | 9 | | 156/153 | 38 | | 153/153 | | 1 | | 48 |  | 48 | 156/0 | 153/0 | |  |
| 4 | 2 | 179/179 | 182/182 | 179/179 | 10 | | 179/182 | 23 | | 182/182 | | 11 | | 44 |  | 44 | 179/0 | 182/0 | |  |
| 4 | 3 | 119/119 | 122/122 | 119/119 | 1 | | 119/122 | 38 | | 122/122 | | 3 | | 42 |  | 42 | 119/0 | 122/0 | |  |
| 4 | 7 | 131/131 | 146/146 | 131/131 | 8 | | 131/146 | 32 | | 146/146 | | 4 | | 44 |  | 44 | 131/0 | 146/0 | |  |
| 4 | 10 | 156/156 | 153/153 | 156/156 | 13 | | 156/153 | 26 | | 153/153 | | 5 | | 44 |  | 44 | 156/0 | 153/0 | |  |
| 5 | 2 | 182/182 | 179/179 | 182/182 | 3 | | 182/179 | 18 | | 179/179 | | 1 | | 22 |  | 22 | 182/0 | 179/0 | |  |
| 5 | 5 | 165/165 | 168/168 | 165/165 | 3 | | 165/168 | 17 | | 168/168 | | 2 | | 22 |  | 22 | 165/0 | 168/0 | |  |
| 5 | 7 | 146/146 | 131/131 |  |  | | 131/146 | 16 | | 146/146 | | 6 | | 22 |  | 22 | 146/0 | 131/0 | |  |
| 5 | 10 | 153/153 | 156/156 | 153/153 | 1 | | 153/156 | 19 | | 156/156 | | 2 | | 22 |  | 22 | 153/0 | 156/0 | |  |
| 6 | 2 | 182/182 | 179/179 | 182/182 | 8 | | 182/179 | 42 | | 179/179 | | 3 | | 53 |  | 53 | 182/0 | 179/0 | |  |
| 6 | 5 | 165/165 | 168/168 | 165/165 | 5 | | 165/168 | 30 | | 168/168 | | 18 | | 53 |  | 53 | 165/0 | 168/0 | |  |
| 6 | 7 | 146/146 | 134/134 | 146/146 | 11 | | 146/134 | 35 | | 134/134 | | 9 | | 55 |  | 55 | 146/0 | 134/0 | |  |
| 6 | 9 | 128/128 | 125/125 | 128/128 | 4 | | 128/125 | 47 | | 125/125 | | 2 | | 53 |  | 53 | 128/0 | 125/0 | |  |
| 7 | 9 | 125/125 | 128/128 | 125/125 | 11 | | 125/128 | 18 | | 128/128 | | 11 | | 40 |  | 40 | 125/0 | 128/0 | |  |
| 8 | 10 | 153/153 | 156/156 | 156/156 | 15 | | 156/153 | 25 | | 153/153 | | 14 | | 54 |  | 54 | 153/0 | 156/0 | |  |
| 9 | 1 | 122/122 | 119/119 | 122/122 | 5 | | 122/119 | 19 | | 119/119 | | 6 | | 30 |  | 30 | 122/0 | 119/0 | |  |
| 9 | 2 | 182/182 | 176/176 | 182/182 | 5 | | 176/182 | 20 | | 176/176 | | 5 | | 30 |  | 30 | 182/0 | 176/0 | |  |
| 9 | 3 | 122/122 | 119/119 | 122/122 | 6 | | 122/119 | 19 | | 119/119 | | 5 | | 30 |  | 30 | 122/0 | 119/0 | |  |
| 9 | 7 | 131/131 | 131/140 | 131/131 | 18 | | 131/140 | 20 | |  | |  | | 38 |  | 38 | 131/131 | 131/140 | |  |
| 9 | 9 | 128/128 | 125/125 | 128/128 | 3 | | 128/125 | 24 | | 125/125 | | 3 | | 30 |  | 30 | 128/0 | 125/0 | |  |
| 9 | 10 | 156/156 | 153/153 | 156/156 | 8 | | 156/153 | 18 | | 153/153 | | 5 | | 31 |  | 31 | 156/0 | 153/0 | |  |
| 10 | 1 | 122/122 | 119/119 |  |  | | 122/119 | 34 | | 119/119 | | 4 | | 38 |  | 38 | 122/0 | 119/119 | |  |
| 10 | 2 | 182/182 | 179/179 | 182/182 | 6 | | 182/179 | 28 | | 179/179 | | 6 | | 40 |  | 40 | 182/0 | 179/0 | |  |
| 10 | 3 | 122/122 | 119/119 | 122/122 | 6 | | 122/119 | 24 | | 119/119 | | 8 | | 38 |  | 38 | 122/0 | 119/0 | |  |
| 10 | 5 | 168/168 | 165/165 | 168/168 | 7 | | 168/165 | 25 | | 165/165 | | 6 | | 38 |  | 38 | 168/0 | 165/0 | |  |
| 10 | 6 | 178/178 | 172/178 | 178/178 | 15 | | 172/178 | 23 | |  | |  | | 38 |  | 38 | 178/178 | 172/178 | |  |
| 10 | 7 | 131/131 | 131/143 | 131/131 | 15 | | 131/143 | 23 | |  | |  | | 38 |  | 38 | 131/131 | 131/143 | |  |
| 10 | 9 | 128/128 | 125/125 |  |  | | 128/125 | 35 | | 125/125 | | 3 | | 38 |  | 38 | 128/0 | 125/125 | |  |
| 10 | 10 | 156/156 | 153/153 | 156/156 | 8 | | 156/153 | 26 | | 153/153 | | 4 | | 38 |  | 38 | 156/0 | 153/0 | |  |
| 11 | 1 | 119/119 | 122/122 |  |  | | 122/119 | 26 | | 119/119 | | 4 | | 30 |  | 30 | 119/0 | 122/0 | |  |
| 11 | 2 | 173/173 | 182/182 | 173/173 | 1 | | 173/182 | 17 | | 182/182 | | 2 | | 20 |  | 20 | 173/0 | 182/0 | |  |
| 11 | 5 | 165/171 | 168/168 | 168/168 | 3 | | 165/168 | 9 | | 168/171 | | 11 | | 23 |  | 23 | 165/171 | 168/168 | |  |
| 11 | 6 | 181/181 | 178/178 | 181/181 | 3 | | 181/178 | 15 | | 178/178 | | 2 | | 20 |  | 20 | 181/0 | 178/0 | |  |
| 12 | 1 | 119/119 | 122/122 |  |  | | 122/119 | 21 | | 119/119 | | 4 | | 25 |  | 25 | 119/0 | 122/0 | |  |
| 12 | 2 | 173/173 | 182/182 | 173/173 |  | | 173/182 | 21 | | 182/182 | | 4 | | 25 |  | 25 | 173/0 | 182/182 | |  |
| 12 | 3 | 122/122 | 119/119 | 122/122 | 19 | |  |  | |  | |  | | 19 | 6 | 25 | 122/0 | 122/122 | |  |
| 12 | 5 | 165/171 | 174/174 | 171/174 | 10 | | 165/174 | 12 | | 174/174 | | 3 | | 25 |  | 25 | 165/171 | 174/174 | |  |
| 12 | 6 | 181/181 | 178/178 |  |  | | 181/178 | 22 | | 178/178 | | 3 | | 25 |  | 25 | 181/0 | 178/178 | |  |
| 12 | 7 | 128/140 | 131/140 | 131/140 | 8 | | 128/140 | 6 | | 128/131 | | 9 | | 23 | 2 | 25 | 128/140 | 131/140 | |  |
| 12 | 9 | 125/125 | 128/128 |  |  | | 125/128 | 22 | | 128/128 | | 3 | | 25 |  | 25 | 125/0 | 128/128 | |  |
| 12 | 10 | 153/153 | 156/156 |  |  | | 156/153 | 22 | | 156/156 | | 3 | | 25 |  | 25 | 153/0 | 156/156 | |  |
| 13 | 1 | 119/119 | 122/122 | 122/122 | 4 | | 122/119 | 22 | | 119/119 | | 4 | | 30 |  | 30 | 119/0 | 122/0 | |  |
| 13 | 2 | 173/173 | 182/182 | 173/173 | 2 | | 173/182 | 26 | | 182/182 | | 2 | | 30 |  | 30 | 173/0 | 182/0 | |  |
| 13 | 5 | 165/171 | 165/165 |  |  | | 165/171 | 15 | | 165/165 | | 15 | | 30 |  | 30 | 165/171 | 165/165 | |  |
| 13 | 6 | 181/181 | 178/178 | 181/181 | 4 | | 181/178 | 20 | | 178/178 | | 6 | | 30 |  | 30 | 181/0 | 178/0 | |  |
| 13 | 7 | 128/140 | 131/131 | 131/140 | 12 | | 128/131 | 15 | | 131/131 | | 3 | | 30 |  | 30 | 128/140 | 131/131 | |  |
| 13 | 9 | 125/125 | 128/128 |  |  | | 125/128 | 28 | | 128/128 | | 2 | | 30 |  | 30 | 125/0 | 128/128 | |  |
| 13 | 10 | 153/153 | 153/153 | 153/153 | 29 | |  |  | |  | |  | | 29 | 2 | 31 | 153/0 | 153/- | |  |
| 14 | 1 | 119/119 | 122/122 | 119/119 | 2 | | 119/122 | 15 | | 122/122 | | 3 | | 20 |  | 20 | 119/0 | 122/0 | |  |
| 14 | 2 | 173/173 | 179/179 | 173/173 | 1 | | 173/179 | 17 | | 179/179 | | 2 | | 20 |  | 20 | 173/0 | 179/0 | |  |
| 14 | 3 | 122/122 | 119/119 | 122/122 | 2 | | 122/119 | 30 | | 119/119 | | 3 | | 35 |  | 35 | 122/0 | 119/0 | |  |
| 14 | 5 | 165/171 | 165/165 |  |  | | 165/171 | 11 | | 165/165 | | 9 | | 20 |  | 20 | 165/171 | 165/0 | |  |
| 14 | 6 | 181/181 | 178/178 |  |  | | 181/178 | 19 | | 178 | | 1 | | 20 |  | 20 | 181/0 | 178/178 | |  |
| 14 | 7 | 128/140 | 143/143 |  |  | | 128/143 | 11 | | 140/143 | | 9 | | 20 |  | 20 | 128/140 | 143/143 | |  |
| 14 | 9 | 125/125 | 125/125 | 125/125 | 18 | |  |  | |  | |  | | 18 | 2 | 20 | 125/0 | 125/125 | |  |
| 14 | 10 | 153/153 | 153/153 | 153/153 | 17 | |  |  | |  | |  | | 17 | 3 | 20 | 153/0 | 153/- | |  |
| 15 | 1 | 122/122 | 119/119 |  |  | | 122/119 | 26 | | 119/119 | | 4 | | 30 |  | 30 | 122/0 | 119/119 | |  |
| 15 | 2 | 182/182 | 179/179 |  |  | | 182/179 | 27 | | 179/179 | | 3 | | 30 |  | 30 | 182/0 | 179/179 | |  |
| 15 | 3 | 122/122 | 119/119 |  |  | | 122/119 | 28 | | 119/119 | | 2 | | 30 |  | 30 | 122/0 | 119/119 | |  |
| 15 | 5 | 168/168 | 165/165 | 168/168 | 3 | | 168/165 | 24 | | 165/165 | | 3 | | 30 |  | 30 | 168/0 | 165/0 | |  |
| 15 | 7 | 131/131 | 131/140 | 131/131 | 15 | | 131/140 | 15 | |  | |  | | 30 |  | 30 | 131/131 | 131/140 | |  |
| 15 | 9 | 128/128 | 125/125 |  |  | | 128/125 | 27 | | 125/125 | | 3 | | 30 |  | 30 | 128/0 | 125/125 | |  |
| 15 | 10 | 156/156 | 153/153 | 156/156 | 8 | | 156/153 | 18 | | 153/153 | | 4 | | 30 |  | 30 | 156/0 | 153/0 | |  |
| 17 | 1 | 119/119 | 122/122 |  |  | | 119/122 | 21 | | 122/122 | | 4 | | 25 |  | 25 | 119/0 | 122/122 | |  |
| 17 | 2 | 179/179 | 179/179 | 179/179 | 23 | |  |  | |  | |  | | 23 | 2 | 25 | 179/0 | 179/179 | |  |
| 17 | 3 | 119/119 | 122/122 |  |  | | 119/122 | 20 | | 122/122 | | 5 | | 25 |  | 25 | 119/0 | 122/122 | |  |
| 17 | 5 | 165/165 | 168/168 |  |  | | 165/168 | 23 | | 168/168 | | 2 | | 25 |  | 25 | 165/0 | 168/168 | |  |
| 17 | 6 | 181/181 | 181/181 | 181/181 | 25 | |  |  | |  | |  | | 25 |  | 25 | 181/0 | 181/181 | |  |
| 17 | 7 | 131/140 | 131/131 |  |  | | 131/140 | 15 | | 131/131 | | 10 | | 25 |  | 25 | 131/140 | 131/131 | |  |
| 18 | 1 | 119/119 | 122/122 | 119/119 | 4 | | 119/122 | 19 | | 122/122 | | 7 | | 30 |  | 30 | 119/0 | 122/0 | |  |
| 18 | 2 | 179/179 | 179/179 | 179/179 | 28 | |  |  | |  | |  | | 28 | 2 | 30 | 179/0 | 179/179 | |  |
| 18 | 3 | 119/119 | 119/119 | 119/119 | 30 | |  |  | |  | |  | | 30 |  | 30 | 119/0 | 119/119 | |  |
| 18 | 5 | 165/165 | 165/165 | 165/165 | 27 | |  |  | |  | |  | | 27 | 3 | 30 | 165/0 | 165/165 | |  |
| 18 | 6 | 181/181 | 178/178 | 181 | 3 | | 181/178 | 22 | | 178/178 | | 5 | | 30 |  | 30 | 181/0 | 178/0 | |  |
| 18 | 7 | 131/140 | 131/140 | 140/140 | 10 | | 131/140 | 14 | | 131/131 | | 6 | | 30 |  | 30 | 131/140 | 131/140 | |  |
| 19 | 1 | 119/119 | 122/122 |  |  | | 119/122 | 42 | | 122/122 | | 6 | | 48 |  | 48 | 119/0 | 122/122 | |  |
| 19 | 2 | 179/179 | 182/182 | 179/179 |  | | 179/182 | 41 | | 182/182 | | 7 | | 48 |  | 48 | 179/0 | 182/182 | |  |
| 19 | 3 | 119/119 | 122/122 |  |  | | 119/122 | 40 | | 122/122 | | 8 | | 48 |  | 48 | 119/0 | 122/122 | |  |
| 19 | 5 | 165/165 | 165/165 | 165/165 | 48 | |  |  | |  | |  | | 48 |  | 48 | 165/0 | 165/165 | |  |
| 19 | 6 | 181/181 | 181/181 | 181/181 | 45 | |  |  | |  | |  | | 45 | 3 | 48 | 181/0 | 181/181 | |  |
| 19 | 7 | 131/140 | 149/149 | 140/149 | 28 | | 131/149 | 20 | |  | |  | | 48 |  | 48 | 131/140 | 149/149 | |  |
| 20 | 1 | 119/119 | 122/122 | 119/119 | 1 | | 119/122 | 27 | | 122/122 | | 2 | | 30 |  | 30 | 119/0 | 122/0 | |  |
| 20 | 2 | 179/179 | 179/179 | 179/179 | 30 | |  |  | |  | |  | | 30 |  | 30 | 179/0 | 179/179 | |  |
| 20 | 3 | 119/119 | 119/119 | 119/119 | 30 | |  |  | |  | |  | | 30 |  | 30 | 119/0 | 119/119 | |  |
| 20 | 5 | 165/165 | 162/162 |  |  | | 165/162 | 26 | | 162/162 | | 3 | | 29 | 1 | 30 | 165/0 | 162/162 | |  |
| 20 | 6 | 181/181 | 172/172 | 181/181 | 2 | | 181/172 | 25 | | 172/172 | | 3 | | 30 |  | 30 | 181/0 | 172/0 | |  |
| 20 | 7 | 131/140 | 131/131 |  |  | | 131/140 | 21 | | 131/131 | | 9 | | 30 |  | 30 | 131/140 | 131/131 | |  |
| 21 | 1 | 125/125 | 122/122 | 125/125 | 1 | | 125/122 | 24 | | 122/122 | | 5 | | 30 |  | 30 | 125/0 | 122/0 | |  |
| 21 | 2 | 179/179 | 182/182 | 179/179 | 2 | | 179/182 | 23 | | 182/182 | | 5 | | 30 |  | 30 | 179/0 | 182/0 | |  |
| 21 | 3 | 122/131 | 119/119 | 131/119 | 12 | | 122/119 | 15 | | 119/119 | | 2 | | 29 | 1 | 30 | 122/131 | 119/119 | |  |
| 21 | 5 | 168/168 | 162/162 |  |  | | 168/162 | 28 | | 162/162 | | 2 | | 30 |  | 30 | 168/0 | 162/162 | |  |
| 21 | 6 | 181/181 | 175/175 |  |  | | 181/175 | 28 | | 175/175 | | 2 | | 30 |  | 30 | 181/0 | 175/175 | |  |
| 21 | 7 | 131/143 | 131/140 | 131/143 | 11 | | 131/140 | 8 | | 131/131 | | 11 | | 30 |  | 30 | 131/143 | 131/140 | |  |
| 21 | 9 | 128/128 | 128/128 | 128/128 | 25 | |  |  | |  | |  | | 25 | 5 | 30 | 128/0 | 128/128 | |  |
| 22 | 1 | 125/125 | 122/122 | 125/125 | 1 | | 125/122 | 24 | | 122/122 | | 5 | | 30 |  | 30 | 125/0 | 122/0 | |  |
| 22 | 2 | 179/179 | 182/182 | 179/179 |  | | 179/182 | 16 | | 182/182 | | 4 | | 20 |  | 20 | 179/0 | 182/182 | |  |
| 22 | 3 | 122/131 | 122/122 |  |  | | 122/131 | 12 | | 122/122 | | 8 | | 20 |  | 20 | 122/131 | 122/122 | |  |
| 22 | 5 | 168/168 | 168/168 | 168/168 | 20 | |  |  | |  | |  | | 20 |  | 20 | 168/0 | 168/168 | |  |
| 22 | 6 | 181/181 | 178/178 |  |  | | 181/178 | 16 | | 178/178 | | 4 | | 20 |  | 20 | 181/0 | 178/178 | |  |
| 22 | 7 | 131/143 | 131/143 | 143/143 | 3 | | 131/143 | 11 | | 131/131 | | 6 | | 20 |  | 20 | 131/143 | 131/143 | |  |
| 22 | 9 | 128/128 | 125/125 |  |  | | 128/125 | 15 | | 125/125 | | 5 | | 20 |  | 20 | 128/0 | 125/0 | |  |
| 23 | 1 | 125/125 | 119/119 |  |  | | 125/119 | 15 | | 119/119 | | 5 | | 20 |  | 20 | 125/0 | 119/119 | |  |
| 23 | 2 | 179/179 | 176/176 | 179/179 |  | | 179/176 | 17 | | 176/176 | | 3 | | 20 |  | 20 | 179/0 | 176/176 | |  |
| 23 | 3 | 122/131 | 119/119 | 131/119 | 9 | | 122/119 | 11 | |  | |  | | 20 |  | 20 | 122/131 | 119/119 | |  |
| 23 | 5 | 168/168 | 165/165 |  |  | | 168/165 | 16 | | 165/165 | | 3 | | 19 | 1 | 20 | 168/0 | 165/165 | |  |
| 23 | 6 | 181/181 | 175/175 |  |  | | 181/175 | 17 | | 175/175 | | 3 | | 20 |  | 20 | 181/0 | 175/175 | |  |
| 23 | 7 | 131/143 | 131/131 |  |  | | 131/143 | 12 | | 131/131 | | 8 | | 20 |  | 20 | 131/143 | 131/0 | |  |
| 23 | 9 | 128/128 | 125/125 |  |  | | 128/125 | 16 | | 125/125 | | 4 | | 20 |  | 20 | 128/0 | 125/125 | |  |
| 24 | 1 | 125/125 | 125/125 | 125/125 | 26 | |  |  | |  | |  | | 26 |  | 26 | 125/0 | 125/125 | |  |
| 24 | 2 | 179/179 | 185/185 | 179/179 |  | | 179/185 | 23 | | 185/185 | | 3 | | 26 |  | 26 | 179/0 | 185/185 | |  |
| 24 | 3 | 122/131 | 122/122 |  |  | | 122/131 | 13 | | 122/122 | | 12 | | 25 | 1 | 26 | 122/131 | 122/122 | |  |
| 24 | 5 | 168/168 | 168/168 | 168/168 | 23 | |  |  | |  | |  | | 23 | 3 | 26 | 168/0 | 168/168 | |  |
| 24 | 6 | 181/181 | 181/181 | 181/181 | 25 | |  |  | |  | |  | | 25 | 1 | 26 | 181/0 | 181/181 | |  |
| 24 | 7 | 131/143 | 131/131 |  |  | | 131/143 | 12 | | 131/131 | | 14 | | 26 |  | 26 | 131/143 | 131/0 | |  |
| 24 | 9 | 128/128 | 125/125 |  |  | | 128/125 | 21 | | 125/125 | | 5 | | 26 |  | 26 | 128/0 | 125/0 | |  |
| 25 | 1 | 128/128 | 125/125 |  |  | | 128/125 | 19 | | 125/125 | | 3 | | 22 |  | 22 | 128/0 | 125/125 | |  |
| 25 | 3 | 125/125 | 128/128 |  |  | | 125/128 | 17 | | 128/128 | | 4 | | 21 | 1 | 22 | 125/0 | 128/128 | |  |
| 25 | 5 | 168/168 | 168/168 | 168/168 | 22 | |  |  | |  | |  | | 22 |  | 22 | 168/0 | 168/168 | |  |
| 25 | 6 | 172/172 | 178/178 |  |  | | 172/178 | 17 | | 178/178 | | 5 | | 22 |  | 22 | 172/0 | 178/178 | |  |
| 25 | 7 | 134/146 | 134/146 | 146/146 | 4 | | 134/146 | 12 | | 134/134 | | 6 | | 22 |  | 22 | 134/146 | 134/146 | |  |
| 26 | 1 | 128/128 | 131/131 |  |  | | 128/131 | 10 | | 128/128 | | 2 | | 12 |  | 12 | 128/0 | 131/131 | |  |
| 26 | 3 | 125/125 | 125/125 | 125/125 | 12 | |  |  | |  | |  | | 12 |  | 12 | 125/0 | 125/125 | |  |
| 26 | 5 | 168/168 | 171/171 |  |  | | 168/171 | 10 | | 171/171 | | 2 | | 12 |  | 12 | 168/0 | 171/171 | |  |
| 26 | 6 | 172/172 | 181/181 |  |  | | 172/181 | 11 | | 181/181 | | 1 | | 12 |  | 12 | 172/0 | 181/181 | |  |
| 26 | 7 | 134/146 | 134/134 |  |  | | 134/146 | 8 | | 134/134 | | 4 | | 12 |  | 12 | 134/146 | 134/134 | |  |
| 27 | 1 | 128/128 | 128/128 | 128/128 | 20 | |  |  | |  | |  | | 20 | 3 | 23 | 128/0 | 128/128 | |  |
| 27 | 3 | 125/125 | 131/131 |  |  | | 125/131 | 20 | | 131/131 | | 3 | | 23 |  | 23 | 125/0 | 131/131 | |  |
| 27 | 5 | 168/168 | 168/168 | 168/168 | 19 | |  |  | |  | |  | | 19 | 4 | 23 | 168/0 | 168/168 | |  |
| 27 | 6 | 172/172 | 181/181 |  |  | | 172/181 | 16 | | 181/181 | | 6 | | 22 | 1 | 23 | 172/0 | 181/181 | |  |
| 27 | 7 | 134/146 | 134/143 | 134/143 | 10 | | 134/146 | 6 | | 134/134 | | 7 | | 23 |  | 23 | 134/146 | 134/143 | |  |
| 28 | 1 | 128/128 | 128/128 | 128/128 | 15 | |  |  | |  | |  | | 15 |  | 15 | 128/0 | 128/128 | |  |
| 28 | 3 | 125/125 | 128/128 |  |  | | 125/128 | 10 | | 128/128 | | 4 | | 14 | 1 | 15 | 125/0 | 128/128 | |  |
| 28 | 5 | 168/168 | 168/168 | 168/168 | 14 | |  |  | |  | |  | | 14 | 1 | 15 | 168/0 | 168/168 | |  |
| 28 | 6 | 172/172 | 187/187 |  |  | | 172/187 | 12 | | 187/187 | | 3 | | 15 |  | 15 | 172/0 | 187/187 | |  |
| 28 | 7 | 134/146 | 134/143 | 134/143 | 1 | | 134/146 | 14 | |  | |  | | 15 |  | 15 | 134/146 | 134/143 | |  |
| 29 | 1 | 128/128 | 119/128 |  |  | | 128/119 | 6 | | 128/128 | | 9 | | 15 | 1 | 16 | 128/0 | 119/128 | |  |
| 29 | 2 | 185/185 | 185/185 | 185/185 | 13 | |  |  | |  | |  | | 13 | 3 | 16 | 185/0 | 185/185 | |  |
| 29 | 5 | 168/168 | 168/168 | 168/168 | 15 | |  |  | |  | |  | | 15 | 1 | 16 | 168/0 | 168/168 | |  |
| 29 | 6 | 187/187 | 181/181 |  |  | | 187/181 | 13 | | 181/181 | | 3 | | 16 |  | 16 | 187/0 | 181/181 | |  |
| 29 | 7 | 137/146 | 134/134 | 134/146 | 6 | | 134/137 | 8 | | 134/134 | | 1 | | 15 | 1 | 16 | 137/146 | 134/134 | |  |
| 29 | 9 | 131/131 | 131/131 | 131/131 | 12 | |  |  | |  | |  | | 12 | 3 | 15 | 131/0 | 131/0 | |  |
| 30 | 1 | 128/128 | 128/128 | 128/128 | 18 | |  |  | |  | |  | | 18 |  | 18 | 128/0 | 128/128 | |  |
| 30 | 2 | 185/185 | 182/182 |  |  | | 185/182 | 16 | | 182/182 | | 2 | | 18 |  | 18 | 185/0 | 182/182 | |  |
| 30 | 5 | 168/168 | 168/168 | 168/168 | 16 | |  |  | |  | |  | | 16 | 2 | 18 | 168/0 | 168/168 | |  |
| 30 | 6 | 187/187 | 190/190 |  |  | | 187/190 | 15 | | 190/190 | | 3 | | 18 |  | 18 | 187/0 | 190/190 | |  |
| 30 | 7 | 137/146 | 134/143 | 134/143 | 2 | | 134/137 | 7 | | 137/143 | | 9 | | 18 |  | 18 | 137/146 | 134/143 | |  |
| 30 | 9 | 131/131 | 131/131 | 131/131 | 17 | |  |  | |  | |  | | 17 | 1 | 18 | 131/0 | 131/0 | |  |
| 31 | 1 | 128/128 | 128/128 | 128/128 | 20 | |  |  | |  | |  | | 20 |  | 20 | 128/0 | 128/128 | |  |
| 31 | 2 | 185/185 | 182/182 |  |  | | 185/182 | 16 | | 182/182 | | 3 | | 19 | 1 | 20 | 185/0 | 182/182 | |  |
| 31 | 5 | 168/168 | 171/171 |  |  | | 168/171 | 18 | | 171/171 | | 2 | | 20 |  | 20 | 168/0 | 171/171 | |  |
| 31 | 6 | 187/187 | 187/187 | 187/187 | 19 | |  |  | |  | |  | | 19 | 1 | 20 | 187/0 | 187/187 | |  |
| 31 | 7 | 137/146 | 134/143 | 137/143 | 7 | | 134/137 | 11 | | 134/143 | | 2 | | 20 |  | 20 | 137/146 | 134/143 | |  |
| 31 | 9 | 131/131 | 134/134 |  |  | | 131/134 | 17 | | 134/134 | | 3 | | 20 |  | 20 | 131/0 | 134/134 | |  |
| 32 | 1 | 128/128 | 125/125 |  |  | | 128/125 | 13 | | 125/125 | | 3 | | 16 |  | 16 | 128/0 | 125/125 | |  |
| 32 | 2 | 185/185 | 179/179 |  |  | | 185/179 | 14 | | 179/179 | | 2 | | 16 |  | 16 | 185/0 | 179/179 | |  |
| 32 | 5 | 168/168 | 168/168 | 168/168 | 16 | |  |  | |  | |  | | 16 |  | 16 | 168/0 | 168/168 | |  |
| 32 | 6 | 187/187 | 181/181 |  |  | | 187/181 | 15 | | 181/181 | | 1 | | 16 |  | 16 | 187/0 | 181/181 | |  |
| 32 | 7 | 137/146 | 134/143 | 137/143 | 5 | | 134/137 | 9 | | 134/146 | | 2 | | 16 |  | 16 | 137/146 | 134/143 | |  |
| 32 | 9 | 131/131 | 131/131 | 131/131 | 16 | |  |  | |  | |  | | 16 |  | 16 | 131/0 | 131/131 | |  |
| 33 | 1 | 125/125 | 128/128 |  |  | | 125/128 | 16 | | 128/128 | | 3 | | 19 | 1 | 20 | 125/0 | 128/128 | |  |
| 33 | 2 | 185/185 | 182/182 |  |  | | 185/182 | 18 | | 182/182 | | 2 | | 20 |  | 20 | 185/0 | 182/182 | |  |
| 33 | 6 | 190/190 | 181/181 |  |  | | 190/181 | 17 | | 181/181 | | 3 | | 20 |  | 20 | 190/0 | 181/181 | |  |
| 33 | 7 | 134/134 | 134/146 | 134/134 | 8 | | 134/146 | 12 | |  | |  | | 20 |  | 20 | 134/134 | 134/146 | |  |
| 33 | 9 | 134/134 | 131/131 |  |  | | 134/131 | 13 | | 131/131 | | 7 | | 20 |  | 20 | 134/134 | 131/0 | |  |
| 33 | 10 | 159/159 | 156/156 |  |  | | 159/156 | 18 | | 156/156 | | 2 | | 20 |  | 20 | 159/0 | 156/156 | |  |
| 34 | 1 | 125/125 | 128/128 |  |  | | 125/128 | 23 | | 128/128 | | 2 | | 25 |  | 25 | 125/0 | 128/128 | |  |
| 34 | 2 | 185/185 | 185/185 | 185/185 | 25 | |  |  | |  | |  | | 25 |  | 25 | 185/0 | 185/185 | |  |
| 34 | 5 | 168/168 | 165/174 | 168/174 | 15 | | 168/165 | 10 | |  | |  | | 25 |  | 25 | 168/168 | 165/174 | |  |
| 34 | 6 | 190/190 | 181/181 |  |  | | 190/181 | 22 | | 181/181 | | 3 | | 25 |  | 25 | 190/0 | 181/181 | |  |
| 34 | 9 | 134/134 | 131/131 |  |  | | 134/131 | 22 | | 131/131 | | 3 | | 25 |  | 25 | 134/134 | 131/0 | |  |
| 34 | 10 | 159/159 | 156/156 |  |  | | 159/156 | 25 | |  | |  | | 25 |  | 25 | 159/159 | 156/156 | |  |
| 35 | 1 | 125/125 | 128/128 |  |  | | 125/128 | 14 | | 128/128 | | 1 | | 15 | 1 | 16 | 125/0 | 128/128 | |  |
| 35 | 2 | 185/185 | 182/182 |  |  | | 185/182 | 15 | | 182/182 | | 1 | | 16 |  | 16 | 185/0 | 182/182 | |  |
| 35 | 6 | 190/190 | 181/181 |  |  | | 190/181 | 14 | | 181/181 | | 1 | | 15 | 1 | 16 | 190/0 | 181/181 | |  |
| 35 | 7 | 134/134 | 134/146 | 134/134 | 9 | | 134/146 | 7 | |  | |  | | 16 |  | 16 | 134/134 | 134/146 | |  |
| 35 | 9 | 134/134 | 131/131 |  |  | | 134/131 | 13 | | 131/131 | | 3 | | 16 |  | 16 | 134/134 | 131/0 | |  |
| 35 | 10 | 159/159 | 156/156 |  |  | | 159/156 | 14 | | 156/156 | | 2 | | 16 |  | 16 | 159/0 | 156/156 | |  |
| 36 | 1 | 125/125 | 128/128 |  |  | | 125/128 | 39 | | 128/128 | | 2 | | 41 | 3 | 44 | 125/0 | 128/128 | |  |
| 36 | 2 | 185/185 | 185/185 | 185/185 | 38 | |  |  | |  | |  | | 38 | 6 | 44 | 185/0 | 185/185 | |  |
| 36 | 3 | 125/125 | 128/128 |  |  | | 125/128 | 39 | | 128/128 | | 5 | | 44 |  | 44 | 125/0 | 128/128 | |  |
| 36 | 5 | 168/168 | 165/165 | 168/168 | 2 | | 168/165 | 36 | | 165/165 | | 6 | | 44 |  | 44 | 168/0 | 165/0 | |  |
| 36 | 6 | 178/178 | 184/184 |  |  | | 178/184 | 38 | | 184/184 | | 5 | | 43 | 1 | 44 | 178/0 | 184/184 | |  |
| 36 | 7 | 137/149 | 134/146 | 146/149 | 11 | | 134/149 | 18 | | 134/137 | | 15 | | 44 |  | 44 | 137/149 | 134/146 | |  |
| 36 | 9 | 131/131 | 134/134 |  |  | | 131/134 | 38 | | 134/134 | | 3 | | 41 | 3 | 44 | 131/0 | 134/134 | |  |
| 36 | 10 | 156/156 | 159/159 |  |  | | 156/159 | 41 | | 159/159 | | 3 | | 44 |  | 44 | 156/0 | 159/159 | |  |
| 37 | 1 | 125/125 | 125/125 | 125/125 | 20 | |  |  | |  | |  | | 20 |  | 20 | 125/0 | 125/125 | |  |
| 37 | 2 | 185/185 | 188/188 | 185/185 | 1 | | 185/188 | 17 | | 188/188 | | 2 | | 20 |  | 20 | 185/0 | 188/188 | |  |
| 37 | 3 | 125/125 | 128/128 | 125/125 | 2 | | 125/128 | 15 | | 128/128 | | 3 | | 20 |  | 20 | 125/0 | 128/128 | |  |
| 37 | 5 | 168/168 | 168/168 | 168/168 | 20 | |  |  | |  | |  | | 20 |  | 20 | 168/0 | 168/168 | |  |
| 37 | 6 | 178/178 | 181/181 | 178/178 | 3 | | 178/181 | 14 | | 181/181 | | 3 | | 20 |  | 20 | 178/0 | 181/0 | |  |
| 37 | 7 | 137/149 | 137/149 | 149/149 | 4 | | 137/149 | 11 | | 137/137 | | 5 | | 20 |  | 20 | 137/149 | 137/149 | |  |
| 37 | 9 | 131/131 | 131/131 | 131/131 | 18 | |  |  | |  | |  | | 18 | 2 | 20 | 131/0 | 131/- | |  |
| 37 | 10 | 156/156 | 156/156 | 156/156 | 20 | |  |  | |  | |  | | 20 |  | 20 | 156/0 | 156/156 | |  |
| 38 | 1 | 125/125 | 128/128 | 125/125 | 1 | | 125/128 | 16 | | 128/128 | | 3 | | 20 |  | 20 | 125/0 | 128/0 | |  |
| 38 | 2 | 185/185 | 179/179 |  |  | | 185/179 | 17 | | 179/179 | | 3 | | 20 |  | 20 | 185/0 | 179/179 | |  |
| 38 | 3 | 125/125 | 125/125 | 125/125 | 18 | |  |  | |  | |  | | 18 | 2 | 20 | 125/0 | 125/125 | |  |
| 38 | 5 | 168/168 | 168/168 | 168/168 | 20 | |  |  | |  | |  | | 20 |  | 20 | 168/0 | 168/168 | |  |
| 38 | 6 | 178/178 | 184/184 |  |  | | 178/184 | 17 | | 184/184 | | 3 | | 20 |  | 20 | 178/0 | 184/184 | |  |
| 38 | 7 | 137/149 | 134/143 | 137/143 | 7 | | 137/134 | 6 | | 149/134 | | 7 | | 20 |  | 20 | 137/149 | 134/143 | |  |
| 38 | 9 | 131/131 | 134/134 |  |  | | 131/134 | 16 | | 134/134 | | 2 | | 18 | 2 | 20 | 131/0 | 134/134 | |  |
| 38 | 10 | 156/156 | 159/159 |  |  | | 156/159 | 17 | | 159/159 | | 3 | | 20 |  | 20 | 156/0 | 159/159 | |  |
| 39 | 1 | 125/125 | 128/128 | 125/125 | 2 | | 125/128 | 16 | | 128/128 | | 2 | | 20 |  | 20 | 125/0 | 128/0 | |  |
| 39 | 2 | 185/185 | 182/182 |  |  | | 185/182 | 16 | | 182/182 | | 4 | | 20 |  | 20 | 185/0 | 182/182 | |  |
| 39 | 3 | 125/125 | 128/128 |  |  | | 125/128 | 18 | | 128/128 | | 2 | | 20 |  | 20 | 125/0 | 128/128 | |  |
| 39 | 6 | 178/178 | 184/184 |  |  | | 178/184 | 17 | | 184/184 | | 3 | | 20 |  | 20 | 178/0 | 184/184 | |  |
| 39 | 7 | 137/149 | 134/146 | 137/146 | 5 | | 137/134 | 8 | | 149/134 | | 7 | | 20 |  | 20 | 137/149 | 134/146 | |  |
| 39 | 9 | 131/131 | 131/131 | 131/131 | 20 | |  |  | |  | |  | | 20 |  | 20 | 131/0 | 131/- | |  |
| 39 | 10 | 156/156 | 156/156 | 156/156 | 19 | |  |  | |  | |  | | 19 | 1 | 20 | 156/0 | 156/156 | |  |
| 17* | 9 | 125/125 | 125/125 | 125/125 | 25 | |  |  | |  | |  | | 25 |  | 25 | 125/- | 125/- | |  |
| 17* | 10 | 153/153 | 153/153 | 153/153 | 25 | |  |  | |  | |  | | 25 |  | 25 | 153/- | 153/- | |  |
| 18* | 9 | 125/125 | 125/125 | 125/125 | 30 | |  |  | |  | |  | | 30 |  | 30 | 125/- | 125/- | |  |
| 18* | 10 | 153/153 | 153/153 | 153/153 | 30 | |  |  | |  | |  | | 30 |  | 30 | 153/- | 153/- | |  |
| 19* | 9 | 125/125 | 125/125 | 125/125 | 48 | |  |  | |  | |  | | 48 |  | 48 | 125/- | 125/- | |  |
| 19* | 10 | 153/153 | 153/153 | 153/153 | 48 | |  |  | |  | |  | | 48 |  | 48 | 153/- | 153/- | |  |
| 2* | 9 | 128/128 | 128/128 | 128/128 | 8 | |  |  | |  | |  | | 8 |  | 8 | 128/- | 128/- | |  |
| 2* | 10 | 156/156 | 153/153 | 156/156 | 4 | |  |  | |  | |  | | 4 | 4 | 8 | 156/- | 153/0 | |  |
| 20* | 9 | 125/125 | 125/125 | 125/125 | 30 | |  |  | |  | |  | | 30 |  | 30 | 125/- | 125/- | |  |
| 20* | 10 | 153/153 | 153/153 | 153/153 | 27 | |  |  | |  | |  | | 27 | 3 | 30 | 153/- | 153/- | |  |
| 21* | 10 | 153/153 | 153/153 | 153/153 | 23 | |  |  | |  | |  | | 23 | 7 | 30 | 153/- | 153/- | |  |
| 22* | 10 | 153/153 | 153/153 | 153/153 | 20 | |  |  | |  | |  | | 20 |  | 20 | 153/- | 153/- | |  |
| 23* | 10 | 153/153 | 153/153 | 153/153 | 20 | |  |  | |  | |  | | 20 |  | 20 | 153/- | 153/- | |  |
| 24* | 10 | 153/153 | 153/153 | 153/153 | 26 | |  |  | |  | |  | | 26 |  | 26 | 153/- | 153/- | |  |
| 25* | 2 | 182/182 | 182/182 | 182/182 | 20 | |  |  | |  | |  | | 20 | 2 | 22 | 182/182 | 182 | |  |
| 25* | 9 | 131/131 | 131/131 | 131/131 | 22 | |  |  | |  | |  | | 22 |  | 22 | 131/- | 131/- | |  |
| 25* | 10 | 156/156 | 156/156 | 156/156 | 22 | |  |  | |  | |  | | 22 |  | 22 | 156/- | 156/- | |  |
| 26* | 2 | 182/182 | 182/182 | 182/182 | 10 | |  |  | |  | |  | | 10 | 2 | 12 | 182/182 | 182 | |  |
| 26* | 9 | 131/131 | 131/131 | 131/131 | 12 | |  |  | |  | |  | | 12 |  | 12 | 131/- | 131/- | |  |
| 26* | 10 | 156/156 | 156/156 | 156/156 | 11 | |  |  | |  | |  | | 11 | 1 | 12 | 156/- | 156/- | |  |
| 27* | 2 | 182/182 | 182/182 | 182/182 | 23 | |  |  | |  | |  | | 23 |  | 23 | 182/182 | 182 | |  |
| 27* | 9 | 131/131 | 131/131 | 131/131 | 23 | |  |  | |  | |  | | 23 |  | 23 | 131/- | 131/- | |  |
| 27* | 10 | 156/156 | 156/156 | 156/156 | 23 | |  |  | |  | |  | | 23 |  | 23 | 156/- | 156/- | |  |
| 28* | 2 | 182/182 | 182/182 | 182/182 | 13 | |  |  | |  | |  | | 13 | 2 | 15 | 182/182 | 182 | |  |
| 28* | 9 | 131/131 | 131/131 | 131/131 | 15 | |  |  | |  | |  | | 15 |  | 15 | 131/- | 131/- | |  |
| 28* | 10 | 156/156 | 156/156 | 156/156 | 15 | |  |  | |  | |  | | 15 |  | 15 | 156/- | 156/- | |  |
| 29* | 3 | 128/128 | 128/128 | 128/128 | 16 | |  |  | |  | |  | | 16 |  | 16 | 128/128 | 128/- | |  |
| 29* | 10 | 156/156 | 156/156 | 156/156 | 16 | |  |  | |  | |  | | 16 |  | 16 | 156/- | 156/- | |  |
| 30* | 3 | 128/128 | 128/128 | 128/128 | 18 | |  |  | |  | |  | | 18 |  | 18 | 128/128 | 128/- | |  |
| 30* | 10 | 156/156 | 156/156 | 156/156 | 18 | |  |  | |  | |  | | 18 |  | 18 | 156/- | 156/- | |  |
| 31* | 3 | 128/128 | 128/128 | 128/128 | 18 | |  |  | |  | |  | | 18 | 2 | 20 | 128/128 | 128/- | |  |
| 31* | 10 | 156/156 | 156/156 | 156/156 | 20 | |  |  | |  | |  | | 20 |  | 20 | 156/- | 156/- | |  |
| 32* | 3 | 128/128 | 128/128 | 128/128 | 16 | |  |  | |  | |  | | 16 |  | 16 | 128/128 | 128/- | |  |
| 32* | 10 | 156/156 | 156/156 | 156/156 | 16 | |  |  | |  | |  | | 16 |  | 16 | 156/- | 156/- | |  |
| 33* | 3 | 128/128 | 128/128 | 128/128 | 19 | |  |  | |  | |  | | 19 | 1 | 20 | 128/128 | 128/- | |  |
| 33* | 5 | 168/168 | 168/168 | 168/168 | 19 | |  |  | |  | |  | | 19 | 1 | 20 | 168/168 | 168/- | |  |
| 34* | 3 | 128/128 | 128/128 | 128/128 | 25 | |  |  | |  | |  | | 25 |  | 25 | 128/128 | 128/- | |  |
| 34* | 7 | 134/134 | 134/134 | 134/134 | 23 | |  |  | |  | |  | | 23 | 2 | 25 | 134/134 | 134/- | |  |
| 35* | 3 | 128/128 | 128/128 | 128/128 | 16 | |  |  | |  | |  | | 16 |  | 16 | 128/128 | 128/- | |  |
| 35* | 5 | 168/168 | 168/168 | 168/168 | 16 | |  |  | |  | |  | | 16 |  | 16 | 168/168 | 168/- | |  |
| 39* | 5 | 168/168 | 168/168 | 168/168 | 17 | |  |  | |  | |  | | 17 | 3 | 20 | 168/0 | 168/- | |  |
| *Excluded from the segregation analysis | | | | | |  |  | |  | |  | |  |  |  |  |  | |  | |
